# Adaptability and stability evaluation of maize hybrids using Bayesian segmented regression models

**DOI:** 10.1371/journal.pone.0236571

**Published:** 2020-07-30

**Authors:** Tâmara Rebecca A. Oliveira, Hélio Wilson L. Carvalho, Moysés Nascimento, Emiliano Fernandes N. Costa, Gustavo Hugo F. Oliveira, Geraldo A. Gravina, Antonio T. Amaral Junior, José Luiz S. Carvalho Filho

**Affiliations:** 1 Núcleo de Graduação de Agronomia, Universidade Federal de Sergipe, Campus Sertão, Nossa Senhora da Glória, Sergipe, Brazil; 2 Embrapa Tabuleiros Costeiros, Aracaju, Sergipe, Brazil; 3 Laboratório de Inteligência Computacional e Aprendizado Estatistico, Departamento de Estatística, Universidade Federal de Viçosa, Viçosa, Brazil; 4 Laboratório de Engenharia Agrícola, Centro de Ciências e Tecnologias Agropecuárias, Universidade Estadual do Norte Fluminense, Rio de Janeiro, Brazil; 5 Departamento de Agronomia, Universidade Federal Rural de Pernambuco, Recife, Brazil; Federal University of Mato Grosso do Sul, BRAZIL

## Abstract

The occurrence of genotype by environment interaction (G x E), which is defined as the differential response of genotypes to environmental variation, is frequently reported in maize cultures, making it challenging to recommend cultivars. Methods allowing to study the potential nonlinear pattern of genotype responses to environmental variation allied to prior beliefs on unknown parameters are interesting to evaluate the phenotypic adaptability and stability of genotypes. In this context, the present study aimed to assess the adaptability and stability of maize hybrids, by using the Bayesian segmented regression model, and evaluate the efficacy of using informative and minimally informative prior distributions for the selection of cultivars. Randomized complete-block design experiments were carried out to study the yield (kg/ha) of 25 maize hybrids, in 22 different environments, in Northeastern Brazil. The Bayesian segmented regression model fitted using informative prior distributions presented lower credibility intervals and Deviance Criterium of Information values, compared to those obtained by fitting using minimally informative distributions. Therefore, the model using informative prior distributions was considered for the adaptability and stability evaluation of maize genotypes. Once most northeastern farmers in Brazil have limited capital, the genotype P4285HX should be considered for planting, due to its high yield performance and adaptability to unfavorable environments.

## Introduction

Maize (*Zea mays* L.) cultures are appreciated worldwide. Thus it has tremendous relevance due to its several uses and applications in areas ranging from animal feed to technological industries.

Furthermore, because maize is grown under different environmental conditions, it interacts with various environments, resulting in varied genotype performances [1]. Such interactions hinder the genotype sealing works given that the best-suited genotype for a specific environment may not be best suited for another environment where such interactions take place. Thus, recommendations for the broad adaptability and stability of cultivars become costly [1].

The literature presents several methodologies to study phenotypic adaptability and stability. Examples of such methods are those based on simple linear regression [2], piecewise linear regression [3, 4], mixed models (REML/BLUP) [5], non-parametric methods [6, 7], multivariate analysis, for example, multiple and expanded centroid methods [8, 9] and Bayesian inference for simple linear regression [10].

Unlike the deterministic and frequentist methods, the Bayesian framework allows for the incorporation of additional information relating to the parameters through prior distributions, which are characterized by the probability distribution. According to [11], all information is useful and must be used in the Bayesian analysis. Additionally, owing to the large quantity of information available from previous studies, incorporating this information during modeling is reasonable [12].

Despite being interesting, the Bayesian approach to adaptability and stability studies is based on a simple regression model [10]. According to [13], the simple linear regression models are unable to fit a potential nonlinear pattern to genotype responses to environmental variations. Aiming to solve this deficiency, under a statistical “frequentist” framework, [3, 4] proposed the segmented regression model allowing the identification of the “ideal” genotype, which presents high yield performance, high stability and low sensitivity to adverse conditions. Nascimento et al. [12] proposed the Bayesian segmented regression model approach to analyze phenotypic adaptability and stability. This approach differs from the “frequentist” framework, allowing the addition of prior beliefs to unknown parameters, bringing new insights for plant breeders. Additionally, this method allows for the exploitation of potential nonlinear patterns of genotype responses to environmental variations, aiming to identify genotypes that present high yield performance, and high stability under adverse conditions. This genotype is denoted as “ideal,” according to [4].

In light of the above, the present study aimed to assess the adaptability and stability of maize hybrids by using the Bayesian segmented regression model and evaluate the efficacy of using informative and minimally informative prior distributions in the selection of cultivars.

## Materials and methods

During the agricultural years 2012 and 2013, 25 maize hybrids from public and private companies from the states of Maranhão (Balsas, Brejo, Colinas, and São Raimundo das Mangabeiras counties), Piauí (Nova Santa Rosa, Teresina, and Uruçuí counties), and Sergipe (Nossa Senhora das Dores, Frei Paulo, and Umbaúba counties) were assessed. The assessments comprised 11 environments, where the Nossa Senhora das Dores County had two different fertilization and each one was assumed as a different environment (Table 1).

**Table 1:** List of hybrid maize cultivars and their respective origins, types, cycles, colors, grain textures and companies.

Cultivar	Transgenic/Conventional	Type^1^	Cycle^2^	Grain color^3^	Grain texture^4^	Company
30A95HX	Transgenic	TH	E	OR	SMHARD	MORGAN
30A68HX	Transgenic	SH	EE	OR	SMHARD	MORGAN
BM820	Conventional	SH	E	R	HARD	BIOMATRIX
DKB330YG	Conventional	SH	EE	R/OR	SMDENT	DEKALB
AS1596R2	Transgenic	SH	E	R	SMDENT	AGROESTE
P4285H	Transgenic	SH	E	Y/OR	HARD	DU PONT
2B710HX	Transgenic	SH	E	Y/OR	SMHARD	DOW
30A16HX	Transgenic	SH	E	OR	SMHARD	MORGAN
DKB370	Conventional	SHm	E	Y/OR	SMHARD	DEKALB
AG8041YG	Transgenic	SH	E	Y/OR	SMHARD	SEMENTES
20A55HX	Transgenic	TH	E	OR	SMHARD	MORGAN
30F53HR	Transgenic	SH	E	OR	SMHARD	DU PONT
30A37HX	Transgenic	SH	EE	Y/OR	SMHARD	MORGAN
30A91HX	Transgenic	SHm	E	Y/OR	SMHARD	MORGAN
2B587HX	Transgenic	SH	E	Y/OR	SMDENT	DOW
2B433HX	Transgenic	TH	EE	Y/OR	SMDENT	DOW
AS1555YG	Transgenic	SH	E	OR	SMHARD	AGROESTE
BRS2022	Conventional	DH	E	OR	SMDENT	EMBRAPA
Statusvip	Transgenic	SH	E	OR	HARD	SYNGENTA
BRS2020	Conventional	DH	E	OR	SMHARD	EMBRAPA
2B707HX	Transgenic	SH	E	OR	SMHARD	DOW
20A78HX	Transgenic	SH	E	OR	SMHARD	DOW
2B604HX	Transgenic	SHm	E	OR	SMHARD	DOW
30K73H	Transgenic	SH	E	Y/OR	SMHARD	DU PONT
2B688HX	Transgenic	TH	E	OR	SMHARD	DOW

^1^DH: Double hybrid; TH: Triple hybrid; SHm: Modified single hybrid.

^2^EE: Extra early; E: Early.

^3^OR: Orange; R: Reddish; Y:Yellow.

^4^SMDENT:Semi-dent; SMHARD:Semi-hard.

During the trials, samples considered to have high fertilization ranges were treated with a total of 180.00 kg ha^-1^ of N, 149.80 kg ha^-1^ of P2O5 and 85.60 kg ha^-1^ of K2O, whereas samples considered to have low fertilization ranges were treated with 45.00 kg ha^-1^ of N, 37.80 kg ha^-1^ of P2O5 and 21.60 kg ha^-1^ of K2O, in the form of 535 and 135 kg ha^-1^ of 8-28-16 Zn at the time of sowing, respectively.

The experimental design was based on randomized blocks, with two repetitions, wherein each plot comprised four 5.0 m-long rows, with spacings of 0.70 m x 0.20 m, between rows, and between holes within the rows, respectively.

Fertilization was performed according to the results of the soil analysis from each experimental area. Irrigation was not carried out, and weed and pest control was performed according to the crop’s requirement in each region.

The maize yield data were subjected to variance analysis for each environment. A joint analysis was carried out by adopting the model: *y_ijk_* = *μ* + *r*/*e_k(j)_* + *e_j_* + *g_i_* + *ge_ij_* + *ε_ijk_*, where *y*_*ijk*_ is the phenotypic mean, *μ* is the overall mean, *r*/*e_k(j)_* is the effect of the *k*^th^ repetition in the *j*^th^ environment, *g*_*i*_ is the fixed effect of the *i*^th^ genotype, *e*_*j*_ is the effect of the *j*^th^ environment normally and independently distributed (NID) $\left(0,\sigma_{e}^{2}\right)$, *ge*_*ij*_ is the effect of the interaction of the ith genotype in the *j*^th^ environment NID $\left(0,\sigma_{ge}^{2}\right)$, and *ε*_*ijk*_ is the experimental error $NID(0,\sigma_{\varepsilon }^{2}).$

### Model and Bayesian inference

The bi-segmented regression model is given by

$$y_{ij}=\beta_{i0}+\beta_{i1}I_{j}+\beta_{i2}T(I_{j})+e_{ij,}$$ (1)

where *y*_*ij*_ is the response of genotype *i* in environment *j*, *β*_*i*0_ is the mean response of genotype *i*, *β*_*i*1_ is the slope under the first regime (the linear regression coefficient related to the unfavorable environments), and *β*_*i*2_ represents the change in slope from the first to the second regime (*β*_*i*1_ + *β*_*i*2_ is the slope after the change-point, that is, the linear response to the favorable environments). Further, *I*_*j*_ is the coded environmental index, *T*(*I*_*j*_) = 0 if *I*_*j*_ ≤ 0, or $T\left(I_{j}\right)=I_{j}-{\bar{I}}_{+}$ if *I*_*j*_ > 0, and ${\bar{I}}_{+}$ is mean of the coded environmental index considering only environments with positive indexes and *e*_*ij*_ is the error term, NID (0, *σ*^2^).

The Bayesian approach for the bi-segmented model is described in Nascimento et al. [12]. In summary, assuming $e_{ij}|I\sigma_{ie}^{2}\sim N(0,I\sigma_{ie}^{2})$, each observation y_ij_ has a distribution ${\text{y}}_{\text{ij}}\sim \text{N}\left(\beta_{\text{i}0}+\beta_{\text{i}1}{\text{I}}_{\text{j}}+\beta_{\text{i}2}\text{T}\left({\text{I}}_{\text{j}}\right);\text{I}\sigma_{\text{ie}}^{2}\right)$, and the likelihood function for each genotype is given by

$$\begin{array}{c} L_{i}(\beta_{i0},\beta_{i1},\beta_{i2},\sigma_{ie}^{2},y_{ij})=\frac{1}{{\left(\sqrt{{2\pi \sigma }_{ie}^{2}}\right)}^{a}}\\ exp\left\{-\frac{1}{{2\sigma }_{ie}^{2}}\sum_{I_{\left\{I_{j\leq 0}\right\}}}{\left[y_{ij}-\beta_{i0}-\beta_{i1}I_{j}\right]}^{2}-\frac{1}{{2\sigma }_{ie}^{2}}\sum_{I_{\left\{I_{j>0}\right\}}}{\left[y_{ij}-\beta_{i0}-\beta_{i1}I_{j}-\beta_{i2}(I_{j}-\overset{-}{I_{+}})\right]}^{2}\right\}. \end{array}$$ (1.1)

The prior distributions for the parameters (*β*_*i*0_, *β*_*i*1_, *β*_*i*2_, $\sigma_{ie}^{2}$) are given by

$$\beta_{i0}⎹\mu_{\beta_{i0}},\sigma_{\beta_{i0}}^{2}\sim N(\mu_{\beta_{i0}},\sigma_{\beta_{i0}}^{2})$$ (1.2)

$$\beta_{i1}⎹\mu_{\beta_{i1}},\sigma_{\beta_{i1}}^{2}\sim N(\mu_{\beta_{i1}},\sigma_{\beta_{i1}}^{2}),$$ (1.3)

$$\beta_{i2}⎹\mu_{\beta_{i2}},\sigma_{\beta_{i2}}^{2}\sim N(\mu_{\beta_{i2}},\sigma_{\beta_{i2}}^{2}),$$ (1.4)

and

$$\frac{1}{\sigma_{ie}^{2}}=\tau_{ie}⎹\alpha_{1},\beta_{1}\sim Gamma(\alpha_{i},\beta_{i}),$$ (1.5)

where μβi0, $\sigma_{\beta_{i0}}^{2}$,μβi1, $\sigma_{\beta_{i1}}^{2}$, μβi2, $\sigma_{\beta_{i2}}^{2}$, and *α*_*i*_, *β*_*i*_ are the known parameters. This last prior distribution is the Gamma distribution with mean and variance equal to $\frac{\alpha }{\beta }$ e $\frac{\alpha }{\beta^{2}}$, respectively. Additionally, _*ie*_ the precision is equal to $\frac{1}{\sigma_{ie}^{2}}$.

The joint posterior distribution is proportional to the product of the likelihood function (Eq 1.1) and the prior distributions (Eqs 1.2–1.5).

P(βi0,βi1,βi2,τei=1σie2|yij)∝1(2πσie2)aexp{−12σie2∑I{Ij≤0}[yij−βi0−βi1Ij]2−12σie2∑I{Ij>0}[yij−βi0−βi1Ij−βi2(Ij−I+¯)]2}−12πσβi02exp[−12σβi02(βi0−μβi0)2]

$$\begin{array}{c} x\frac{1}{\sqrt{2\pi \sigma_{\beta_{i1}}^{2}}}\text{exp}\left[-\frac{1}{2\sigma_{\beta_{i1}}^{2}}{\left(\beta_{i1}-\mu_{\beta_{i1}}\right)}^{2}\right]\\ x\frac{1}{\sqrt{2\pi \sigma_{\beta_{i2}}^{2}}}\text{exp}\left[-\frac{1}{2\sigma_{\beta_{i2}}^{2}}{\left(\beta_{i2}-\mu_{\beta_{i2}}\right)}^{2}\right]\\ x\frac{\beta_{i}^{\alpha 1}\tau_{ie}^{\alpha_{1}-1}e^{\beta_{i}\tau_{ei}}}{Г\left(\alpha_{i}\right)} \end{array}$$ (2)

To make inferences regarding the parameters in Eq 2, the Markov chain Monte Carlo (MCMC) was used to obtain the posterior marginal distributions for each parameter.

The marginal distribution samples of the stability parameter, $\sigma_{di}^{2}$, were obtained indirectly. This parameter is a function of $\sigma_{ie}^{2}$. Therefore, using the $\sigma_{ie}^{2}$ values from each interaction, we obtain $\sigma_{di}^{2}$ according to the following expression: ${\hat{\sigma }}_{di}^{2}={\hat{\sigma }}_{ie}^{2}-\frac{MSR}{r}$, where MSR is the residual mean square obtained from the variance analysis and *r* is the number of repetitions of the experiment. The hypotheses of interest were tested by calculating the 95% credibility intervals for the parameters.

### *Priors* distributions

Two models were fitted to assess the model’s goodness of fit. Model 1 (M1—minimally informative prior distributions) was characterized by minimally informative prior distributions, which were represented by distributions with large variances: βi0|μ,βi0σβi02~N(μ=βi00,σβi02=100000),βi1|μ,βi1σβi12~N(μ=βi10,σβi12=100000), βi2|μ,βi2σβi22~N(μ=βi20,σβi22=100000), and *τ*_*ie*_|*α*_*i*_, *β*_*i*_ ~ *Gamma*(*α*_*i*_ = 0.001, *β*_*i*_ = 0.001).

Model 2 (M2—informative prior distributions), similar to the method employed in [12], was characterized by the estimates obtained from the frequentist analysis of the bi-segmented model, used as information to define the hyperparameters.

### Assessing the model’s goodness of fit

Models M1 (minimally informative priors) and M2 (informative priors) were compared by means of the Deviance Information Criterion (DIC) [14]: $DIC=D\left(\hat{\theta }\right)-2p_{D}$. Here, $D\left(\hat{\theta }\right)$ is a point estimate of the deviance obtained by replacing the parameters with their posterior mean estimates in the likelihood function and 2*p*_*D*_ is given by the effective number of parameters in the models. Models with lower DIC are preferred.

### Bayesian analysis

We adopted MCMC chains considering 100,000 iterations of the Gibbs sampler algorithm. We set the burn-in to 10,000 iterations and thinned every five iterations. In each chain, we analyzed the posterior mean, standard deviation, 95% credibility intervals, and convergence criterion statistics [15, 16]. The methodology was implemented in software R [17], and the joint distribution samples were obtained using the rbugs function of the rbugs package [18], which was accomplished by fusing R and OpenBugs (a software application for the Bayesian analysis of complex statistical models using MCMC methods). The MCMC chain convergence was accessed by Geweke and Raftery-Lewis diagnostics using the package [19] provided in the R software [17].

## Results and discussion

The analysis of variance of the maize yield (kg/ha) demonstrated that the genotypes, environments and the genotype x environment interaction (G×E) presented a significant effect (P < 0.05) (Table 2). The significance of G x E interaction indicates contrasts between environments and differential genotypic responses to environmental effects. The occurrence of G × E interaction, which can be defined as the differential response of genotypes to environmental variation, is frequently reported in maize cultures, making it challenging to recommend cultivars [20–24].

**Table 2:** Mean squares from analysis of variance (ANOVA) for yield of 25 hybrid maize genotypes assessed in 22 environments.

Sources of variation	DF	Mean square (MS)
Blocks/Environments	22	1989297
Genotypes (G)	24	16241624**
Environments (E)	21	114452906**
Genotypes x Environments (G x E)	504	2454148**
Error	528	708661
Mean (kg/ha)		8682.99

**Significant at 0.01 probability levels by F test.

The posteriori means and their respective credibility intervals (CI) provided estimates for the adaptability and stability parameters. Considering the results provided by the model M1, which is characterized by the minimally informative prior distributions, most genotypes (14 genotypes) presented the linear regression coefficient related to the unfavorable environments equal to 1 (*β*_*i*1_ = 1), except 30A16HX, 2B707HX, 2B587HX, 30A37HX, 2B604HX, 20A55HR, 20A78HX and DKB370, which presented values higher than 1 (*β*_*i*1_ > 1), and the genotypes P4285HX and BRS2020, which presented values lower than 1 (*β*_*i*1_ < 1) (Table 3). Among those genotypes that presented the linear regression coefficient related to the unfavorable environments equal to 1, only two (30A68HX and AS1555YG) exhibited the linear response to the favorable environments higher than 1 (*β*_*i*1_ + *β*_*i*2_ > 1) (Table 3). No genotype presented stability parameter ($\sigma_{di}^{2}$) equal to zero. On the other hand, the genotype AS1555YG presented coefficient of determination higher than 80% (Table 3). However, only the genotype 30A68HX presented higher mean productivity (${\hat{\beta }}_{0;30\text{A}68\text{HX}}=9465.56>\hat{\mu }=8682.99$).

**Table 3:** Estimates of the *a posteriori* means (${\overset{-}{\beta }}_{0i}$) and of the credible intervals^1^ (95%) of the adaptability^2^ (${\overset{-}{\beta }}_{1i}\;\text{and}\;{\overset{-}{\beta }}_{1i}+{\overset{-}{\beta }}_{2i}$) and stability parameters^3^ (${\overset{-}{\sigma }}_{di}^{2}$, R^2^), by taking into consideration informative and non-informative priors for maize hybrids.

**Genotypes**	$LI{\overset{-}{\beta }}_{0i}$	${\overset{-}{\beta }}_{0i}$	$LS{\overset{-}{\beta }}_{0i}$	$LI{\overset{-}{\beta }}_{1i}$	${\overset{-}{\beta }}_{1i}$	$LS{\overset{-}{\beta }}_{1i}$	$LI{\overset{-}{\beta }}_{1i}+{\overset{-}{\beta }}_{2i}$	${\overset{-}{\beta }}_{1i}+{\overset{-}{\beta }}_{2i}$	$LS{\overset{-}{\beta }}_{1i}+{\overset{-}{\beta }}_{2i}$	$LI{\overset{-}{\sigma }}_{di}^{2}$	${\overset{-}{\sigma }}_{di}^{2}$	$LS{\overset{-}{\sigma }}_{di}^{2}$	**R**^**2**^
	**Minimally informative priors (M1)**												
30A68HX	9316.00	9465.56	9911.00	0.79	0.90	1.24	1.53	1.79	2.58	841275.00	1115511.90	2114100.00	77.44
30A16HX	9295.00	9431.87	9838.03	1.44	1.54	1.85	1.72	1.95	2.67	701600.00	930323.50	1763075.00	89.85
2B707HX	9134.00	9288.44	9748.03	1.05	1.17	1.52	1.17	1.44	2.25	896975.00	1189343.80	2254100.00	80.12
2B587HX	9088.00	9200.56	9536.00	1.24	1.32	1.58	1.26	1.45	2.04	477375.00	632954.50	1199075.00	90.03
30A37HX	9072.00	9195.19	9560.03	1.14	1.23	1.51	0.75	0.96	1.61	566600.00	751357.90	1424050.00	86.00
2B604HX	9052.00	9194.07	9615.03	1.02	1.13	1.45	1.57	1.82	2.56	752675.00	998071.40	1891100.00	83.58
2B710HX	9067.00	9181.73	9524.00	0.98	1.07	1.33	0.80	1.00	1.60	496300.00	658055.00	1247050.00	84.56
30A95HX	8894.00	9075.28	9615.03	1.08	1.22	1.63	1.17	1.48	2.43	1237000.00	1640324.80	3108150.00	76.18
P4285HX	8915.00	9055.94	9474.03	0.52	0.62	0.94	0.79	1.03	1.77	743475.00	985850.50	1868100.00	63.06
30F53HR	8816.00	9009.92	9585.03	0.74	0.88	1.32	-0.16	0.18	1.19	1404000.00	1862281.10	3529150.00	56.08
2B433HX	8871.75	8960.69	9226.00	0.97	1.04	1.24	0.79	0.95	1.42	298500.00	395843.90	750037.50	89.45
20A55HR	8733.00	8834.60	9136.00	1.14	1.22	1.45	0.56	0.73	1.26	385100.00	510603.50	967545.00	89.34
20A78HX	8652.00	8817.59	9311.03	1.06	1.18	1.56	0.59	0.88	1.75	1034000.00	1370711.40	2597125.00	75.88
DKB370	8610.00	8759.79	9184.00	1.11	1.21	1.53	1.24	1.49	2.27	788600.00	1055663.80	2012125.00	83.03
2B688HX	8612.00	8757.44	9190.00	0.82	0.93	1.26	0.31	0.56	1.32	792775.00	1051201.20	1992100.00	71.56
AG8041YG	8601.00	8744.84	9173.03	0.73	0.84	1.17	0.56	0.81	1.57	779900.00	1034193.00	1960075.00	69.57
30K73H	8578.00	8732.15	9190.00	0.98	1.09	1.44	0.79	1.06	1.86	888300.00	1177882.70	2232100.00	76.85
DKB330	8295.00	8475.94	9015.03	0.90	1.03	1.44	0.81	1.12	2.07	1233000.00	1634763.20	3098125.00	69.38
AS1596YG	8200.00	8363.82	8851.00	0.73	0.85	1.22	0.80	1.08	1.94	1006000.00	1333766.70	2527125.00	66.98
BM820	8140.00	8269.68	8654.00	0.90	1.00	1.29	-0.27	-0.04	0.63	626600.00	830832.00	1574075.00	77.21
AS1555YG	7877.00	7996.51	8365.03	0.65	0.74	1.02	1.67	1.88	2.54	580050.00	777663.90	1477075.00	80.68
Statusvip	7495.00	7780.31	8629.03	0.75	0.96	1.61	0.42	0.92	2.41	3056000.00	4052797.70	7680325.00	46.09
BRS2022	7453.00	7576.31	7944.03	0.63	0.72	1.00	0.09	0.30	0.95	574200.00	761371.30	1443050.00	67.12
30A91HX	7293.00	7487.64	8067.00	0.42	0.56	1.00	-0.40	-0.07	0.96	1424000.00	1887726.00	3577175.00	37.25
BRS2020	7346.00	7466.96	7826.03	0.40	0.49	0.76	-0.17	0.04	0.67	547575.00	726058.60	1376050.00	50.86
**Informative priors (M2)**													
30A68HX	9464.00	9464.00	9464.00	0.81	0.90	1.17	1.59	1.80	2.41	679400.00	863019.30	1503000.00	76.90
30A16HX	9430.00	9430.00	9430.00	1.46	1.54	1.80	1.77	1.96	2.53	576100.00	731857.10	1274025.00	89.75
2B707HX	9287.00	9287.00	9287.00	1.08	1.17	1.45	1.24	1.45	2.07	720600.00	915214.00	1593025.00	79.60
2B587HX	9199.00	9199.00	9199.00	1.25	1.32	1.54	1.29	1.46	1.95	409675.00	520498.60	907202.50	90.00
30A37HX	9194.00	9194.00	9194.00	1.16	1.23	1.46	0.79	0.97	1.49	475900.00	604773.60	1054000.00	85.78
2B604HX	9193.00	9192.79	9193.00	1.04	1.13	1.39	1.63	1.82	2.41	613900.00	779879.20	1358000.00	83.29
2B710HX	9180.00	9180.33	9181.00	0.99	1.07	1.29	0.84	1.01	1.50	423700.00	538376.70	937920.00	84.36
30A95HX	9073.00	9073.00	9073.00	1.11	1.22	1.54	1.25	1.49	2.19	971375.00	1233075.60	2145000.00	75.36
P4285HX	9054.00	9054.01	9054.00	0.54	0.62	0.88	0.84	1.04	1.62	607100.00	771220.60	1343000.00	61.97
30F53HR	9008.00	9008.00	9008.00	0.77	0.88	1.22	-0.06	0.19	0.92	1095000.00	1388961.00	2415000.00	53.95
2B433HX	8960.00	8960.00	8960.00	0.98	1.04	1.22	0.81	0.95	1.37	276400.00	351203.30	612807.50	89.50
20A55HR	8833.00	8833.58	8834.00	1.15	1.22	1.41	0.58	0.74	1.19	340900.00	433232.50	755505.00	89.29
20A78HX	8816.00	8816.00	8816.00	1.08	1.18	1.48	0.67	0.89	1.55	821775.00	1043252.90	1815025.00	75.06
DKB370	8756.00	8756.00	8756.00	0.85	0.93	1.20	0.37	0.57	1.16	643500.00	817506.60	1424000.00	70.66
2B688HX	8755.00	8755.00	8755.00	1.12	1.21	1.48	1.29	1.49	2.09	649075.00	824492.50	1436000.00	82.39
AG8041YG	8743.00	8743.00	8743.00	0.76	0.84	1.11	0.62	0.82	1.41	634000.00	805464.40	1403000.00	68.65
30K73H	8730.00	8730.26	8731.00	1.00	1.10	1.37	0.86	1.07	1.69	714275.00	907119.60	1579025.00	76.18
DKB330	8474.00	8474.00	8474.00	0.93	1.04	1.35	0.89	1.13	1.83	968275.00	1229178.30	2138000.00	68.21
AS1596YG	8362.00	8362.00	8362.00	0.76	0.86	1.15	0.87	1.09	1.74	801200.00	1017188.20	1770000.00	65.83
BM820	8268.00	8268.00	8268.00	0.92	1.00	1.24	-0.22	-0.04	0.51	520500.00	661250.80	1152025.00	76.53
AS1555YG	7990.00	7990.44	7991.00	0.67	0.74	0.98	1.70	1.88	2.41	496000.00	630259.80	1099000.00	80.08
Statusvip	7777.00	7777.00	7777.00	0.82	0.97	1.41	0.61	0.93	1.86	2302000.00	2913389.60	5059000.00	42.69
BRS2022	7575.00	7575.00	7575.00	0.64	0.72	0.95	0.13	0.31	0.84	481600.00	611894.50	1067000.00	66.20
30A91HX	7485.00	7485.65	7486.00	0.45	0.56	0.90	-0.30	-0.06	0.68	1109000.00	1406803.70	2445025.00	33.92
BRS2020	7466.00	7466.00	7466.00	0.42	0.49	0.72	-0.13	0.04	0.56	461800.00	586780.90	1022025.00	49.27

^1^LI: Lower Bound; LS: Upper Bound.

^2${\overset{-}{\beta }}_{1i}$^: is the linear regression coefficient related to the unfavorable environments; ${\overset{-}{\beta }}_{1i}+{\overset{-}{\beta }}_{2i}$: is the linear response to the favorable environments.

^3^ ${\overset{-}{\sigma }}_{di}^{2}$: is the stability parameter; R^2^: is the coefficient of determination.

Considering the results provided by the model M1, which is characterized by the minimally informative prior distributions, most genotypes (14 genotypes) presented the linear regression coefficient related to the unfavorable environments equal to 1 (*β*_*i*1_ = 1), except 30A16HX, 2B707HX, 2B587HX, 30A37HX, 2B604HX, 20A55HR, 20A78HX and DKB370, which presented values higher than 1 (*β*_*i*1_ > 1) and the genotypes P4285HX and BRS2020, which presented values lower than 1 (*β*_*i*1_ < 1) (Table 3). Among those genotypes that presented the linear regression coefficient related to the unfavorable environments equal to 1, only two (30A68HX and AS1555YG) presented the linear response to the favorable environments higher than 1 (*β*_*i*1_ + *β*_*i*2_ > 1) (Table 3). No genotype presented stability parameter ($\sigma_{di}^{2}$) equal to zero. On the other hand, the genotype AS1555YG presented coefficient of determination higher than 80% (Table 3). However, only the genotype 30A68HX presented higher mean productivity (${\hat{\beta }}_{0;30\text{A}68\text{HX}}=9465.56>\hat{\mu }=8682.99$).

According to the results obtained by Model 2 (M2), which is characterized by the informative prior distributions, out of the 25 genotypes, 11 (30A68HX, 2B710HX, 30F53HR, 2B433HX, DKB370, AG8041YG, 30K73H, DKB330, AS1596YG and BM820) presented the linear regression coefficient related to the unfavorable environments equal to 1 (*β*_*i*1_ = 1). The nine genotypes observed in the previous analysis presented values higher than 1 (*β*_*i*1_ > 1) and five (P4285HX, AS1555YG, BRS2022, 30A91HX and BRS2020) presented values lower than 1 (*β*_*i*1_ < 1) (Table 3). Among those genotypes that presented the linear regression coefficient related to the unfavorable environments equal to 1, only the genotype 30A68HX showed the linear response to the favorable environments higher than 1 (*β*_*i*1_ + *β*_*i*2_ > 1) and high mean productivity (${\hat{\beta }}_{0;30\text{A}68\text{HX}}=9465.56>\hat{\mu }=8682.99$) (Table 3). This genotype is suitable for growers who employ high-level technology, since it responds well to improved environments [13]. In line with the M1 results, the genotype 30A68HX presented stability parameter ($\sigma_{di}^{2}$) higher than zero and coefficient of determination lower than 80% (Table 3).

The analysis considering M2 was able to better discriminate the genotypes, since, out of 25 genotypes, 14 and 11 presented the linear regression coefficient related to the unfavorable environments equal to 1 for M1 and M2 fitted models, respectively.

A comparative analysis of the limits of credibility intervals obtained by the two fitted models (M1 and M2) reveals that the use of informative prior distributions (M2) reduced the limits of credibility intervals, when compared to minimally informative prior distributions (M1). Similar results were observed by [11], who used the Bayesian segmented regression model for adaptability and stability evaluation of cotton genotypes. Nascimento et al. [10], Couto et al. [20] and Teodoro et al. [25] used the Eberhart and Russel’s Bayesian method to evaluate the phenotypic stability and adaptability of alfalfa and popcorn cultivars and obtained similar results. Additionally, the difference in DIC values between models using minimally informative and informative priors ranged between 1.59 and 2.01. Once smaller DIC values indicate better data fitting, these results demonstrate that M2 should be considered for the adaptability and stability evaluation of maize genotypes (Table 4).

**Table 4:** Deviance Information Criterion (DIC) values obtained through the difference of DIC values between models using minimally informative (i) and informative priors (j) for maize hybrids.

Genotypes	*Dic*_*ij*_
30A68HX	1.80
30A16HX	1.78
2B707HX	1.80
2B587HX	1.75
30A37HX	1.77
2B604HX	1.79
2B710HX	1.76
30A95HX	1.84
P4285H	1.79
30F53HR	1.85
2B433HX	1.65
20A55HX	1.72
20A78HX	1.82
DKB370	2.00
2B688HX	1.59
AG8041YG	1.79
30K73H	1.80
DKB330YG	1.84
AS1596R2	1.81
BM820	1.78
AS1555YG	1.71
Statusvip	2.01
BRS2022	1.77
30A91HX	1.86
BRS2020	1.77

Overall, the Bayesian framework of the segmented regression model allowed the incorporation of additional information related to the parameters, through prior distributions, which reduced the ranges of the credibility intervals, increased the precision of parameter estimates, and, consequently, provided reliable genotype selection. In practice, this information can be obtained from previous studies, including [10, 20]. Due to the lack of prior information related to the evaluated maize hybrids in the literature, in this work, the estimates obtained from the frequentist analysis of the segmented model were used to define the hyperparameters.

In practice, most northeastern farmers in Brazil have limited capital, which prevents them from investing in production technology. Therefore, genotypes adapted to unfavorable environments should be considered for low technology planting [26]. The recommendation of cultivars not adapted to regional conditions leads to low yield and other serious problems, such as the indiscriminate use of pesticides and excessive cultural treatment [27]. Considering the results provided by the model M2 (informative prior distributions), only the genotype P4285HX presented the linear regression coefficient related to the unfavorable environments lower than 1 (*β*_*i*1_ < 1) and high mean productivity (${\hat{\beta }}_{0;\text{P}4285\text{HX}}=9054.01>\hat{\mu }=8682.99$) (Table 3).

## Conclusions

Incorporating additional information about the parameters through prior distributions decreases the credibility interval ranges. The difference in DIC values between models using minimally informative (M1) and informative priors (M2) was positive, which indicates a better data fitting, considering M2. Therefore, it should be an alternative for the adaptability and stability evaluation of maize genotypes.

The genotype P4285HX presents high yield performance and adaptability to unfavorable environments and should be considered for low technology planting, which is practiced by northeastern Brazilians farmers.

## Supporting information

S1 Data (XLSX)
